# Efficacy of a New Commercial Vaccine Against *Clostridioides difficile* and *Clostridium perfringens* Type A for Recurrent Swine Neonatal Diarrhea Under Field Conditions

**DOI:** 10.3390/ani15091200

**Published:** 2025-04-23

**Authors:** Ainhoa Puig Ambrós, Gabriel Peixoto Faria, Massimiliano Baratelli, Roberto Maurício Carvalho Guedes, Rodrigo Otávio Silveira Silva, Oriol Boix-Mas, Xavier Gibert

**Affiliations:** 1 Laboratorios HIPRA, S.A. Avinguda de la Selva, 135, 17170 Amer, Girona, Spain; gabriel.faria@hipra.com (G.P.F.); massimiliano.baratelli@hipra.com (M.B.); oriol.boix@hipra.com (O.B.-M.); xavier.gibert@hipra.com (X.G.); 2 Veterinary School, Federal University of Minas Gerais, Av. Antônio Carlos, Pampulha 6627, Belo Horizonte, Brazil; guedesufmg@gmail.com (R.M.C.G.); rodrigo.otaviosilva@gmail.com (R.O.S.S.)

**Keywords:** vaccine efficacy, *Clostridioides difficile*, *Clostridium perfringens* type A, neonatal diarrhea, swine, weight performance, clinical trial, antimicrobial drug use

## Abstract

Neonatal diarrhea causes significant economic losses in swine production by reducing average daily weight gain (ADWG) and increasing piglet mortality, with *Clostridioides difficile* (CD) and *Clostridium perfringens* type A (CPA) being the most common causes. The aim of this study was to evaluate the efficacy of a new commercial vaccine against these agents to minimize diarrhea, pre-weaning mortality, and its negative consequences on weight performance in suckling piglets under field conditions. The study consisted of two randomized, double-blind, negative-controlled field trials (Study A and B) focusing on clinically healthy pregnant sows from commercial pig farms experiencing recurrent neonatal diarrhea. In the meta-analysis of both farms, the control group showed lower performance compared to the vaccine group (least squares means differences) for ADWG (−14.5 g/day, *p* < 0.001), body weight (−0.33 kg, *p* < 0.001), and underweight piglets at weaning (6.94%, *p* = 0.011). The number of piglets with diarrhea (9.76%, *p* < 0.001) and the percentage of piglets treated with antibiotics for diarrhea (6.09%, *p* = 0.016) were lower in vaccinated animals compared to controls. No significant differences in pre-weaning mortality were observed. The results of this study suggest that the new commercial vaccine against CD and CPA reduces the incidence of neonatal diarrhea and the associated use of antibiotics, while positively impacts the growth performance of suckling piglets.

## Simple Summary

Neonatal diarrhea in piglets, caused by *Clostridioides difficile* and *Clostridium perfringens* type A, leads to economic losses in pig production by reducing growth rates and increasing piglet mortality and treatment costs. This study aimed to test a new commercial vaccine against these bacteria to reduce diarrhea and improve piglet growth. The research was conducted on two farms with recurring diarrhea outbreaks and showed that vaccinating pregnant sows produced piglets with higher growth rates, greater body weight, and fewer underweight at weaning. No differences in pre-weaning mortality were detected. It also led to a lower incidence of diarrhea in piglets and reduced the need for antibiotic treatments. Thus, the vaccine offers valuable benefits to farmers by reducing economic losses and promoting better animal health.

## 1. Introduction

Neonatal diarrhea causes significant economic losses in swine production by reducing the average daily weight gain (ADWG) and increasing piglet mortality, especially in the absence of treatment [1,2,3]. The most frequent agents involved in swine neonatal diarrhea include viruses (e.g., rotaviruses, coronaviruses) and bacteria such as *Escherichia coli* (EC), *Clostridioides difficile* (CD), and *Clostridium perfringens* type A (CPA) and type C (CPC) [3].

*Clostridioides difficile* stands out within the major enteropathogens in piglets due to its high prevalence in some countries [4]. It affects piglets up to 7 days of age, causing a reduction of 0.45–0.9 kg per pig at weaning [4,5,6]. In addition to its importance as an enteropathogen in piglets, CD is possibly a zoonotic agent [7,8,9]. In this context, effective control of CD infection in piglets not only holds direct implications on swine production but also plays a crucial role in the broader epidemiological landscape from a One Health perspective [7,8,9].

*Clostridium perfringens* can produce at least 20 known toxins, six of which are used to classify this bacterium into seven toxinotypes (types A, B, C, D, E, F, and G) [10,11]. CPC infection can cause fatal necrohemorrhagic enteritis, mostly in suckling piglets, and the disease usually presents in 3-day-old piglets [12]. In addition, CPA and its alpha toxin are frequently found in the intestinal tract of healthy animals [12], but this bacterium has also been associated with diarrhea in piglets aged from 1 to 7 days [13].

Given the economic impact of neonatal diarrhea on the swine industry [14] and its infectious etiology in most cases [15], there is a need for adopting preventive strategies such as a good vaccination plan. The vaccination of pregnant sows is a powerful tool for controlling neonatal infections in piglets through passive immunization [16,17]. Recently, the first commercial vaccine against CD infection and CPA in piglets became available, demonstrating proven effects in reducing the incidence of diarrhea and antibiotic use [18]. Thus, the aim of this study was to evaluate the efficacy of this new vaccine in minimizing its negative impact on weight performance and pre-weaning mortality, as well as to confirm its effect on diarrhea and antibiotic use in suckling piglets under field conditions.

## 2. Materials and Methods

### 2.1. Study Animals, Experimental Design, and Exclusion Criteria

This study consisted of two randomized, double-blind, negative-controlled field trials (Study A and B) focusing on clinically healthy pregnant sows from commercial pig farms experiencing recurrent neonatal diarrhea. Study A took place on a farm in Zaragoza (Spain) in 2021, and study B occurred on a farm in Minas Gerais (Brazil) in 2021. Study A encompassed two consecutive batches of 27 sows each, while Study B involved a single batch of 54 sows. Sows were allocated randomly at a 1:1 ratio into either the test vaccine group or the control group, stratified by parity. Cross-fostering between sows of the same group was allowed. Weaning was performed at 24 days (Study A) and 21 days (Study B) after farrowing.

Study A was performed in a breeder multiplier farm, where the genetic lines were TOPIC and PIC, and with 715 sows and a mean (SD) number of liveborn piglets per sow of 14.4 (3.9). All sows were already vaccinated against EC and CPC (Suiseng^®^ Coli/C, HIPRA, Amer, Girona, Spain). Study B was performed in a farrow-to-finish farm with 5300 breeders from genetic lines Danbred and PIC, a mean (SD) number of liveborn piglets per sow of 12.4 (3.5). All sows were already vaccinated against EC and CPC using a commercial vaccine (Porcilis^®^ ColiClos, Merck Sharp and Dohme, Rahway, NJ, USA) and with an autogenous vaccine against CPA and EC F4, F18.

**Figure 1:**
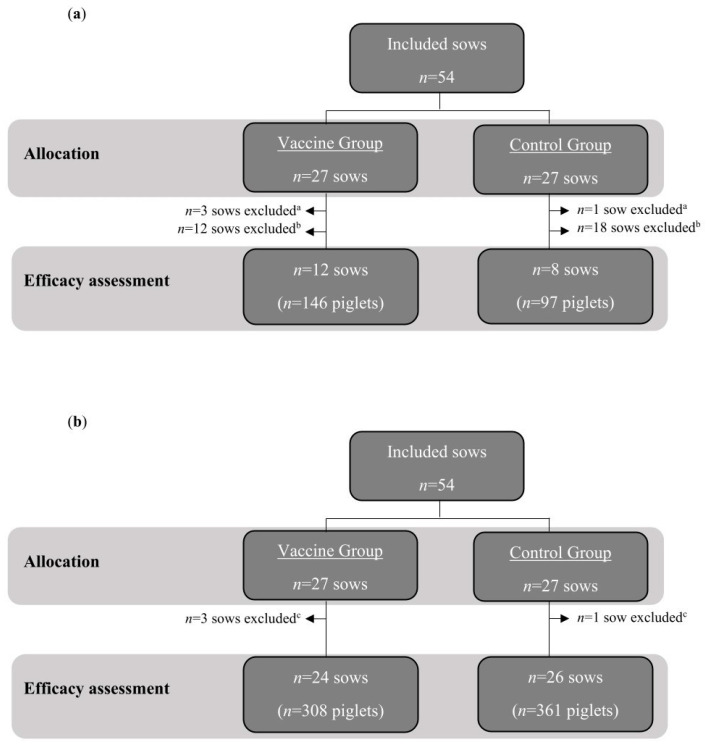
Study population and allocation into treatment groups in studies A (**a**) and B (**b**). ^a^ Dying at the last third of gestation or not giving birth to any living piglet. ^b^ Missing data on monitoring of their piglets’ diarrhea. ^c^ Aborted (*n* = 2), did not farrow any living piglets (*n* = 1), or farrowed prematurely (*n* = 1).

A total of 34 sows from Study A and four from Study B were excluded following the exclusion criteria set in each study protocol (i.e., gestation/farrowing issues and missing data). Therefore, the assessment was conducted on 243 piglets, farrowed by 20 sows in Study A (Figure 1a) and 669 piglets from 50 sows in Study B (Figure 1b).

### 2.2. Commercial Vaccine

The test vaccine (Suiseng^®^ Diff/A, HIPRA, Amer, Girona, Spain) consisted of toxoid alpha of CPA and toxoids A and B of CD as active substances, and aluminum hydroxide, ginseng extract, and diethylaminoethyl-dextran, as adjuvants. The vaccine was administered according to the specified doses, alongside the EC and CPC vaccine (Suiseng^®^ Coli/C, HIPRA, Amer, Girona, Spain) (4 mL), following the schedules outlined in the summary of product characteristics. Specifically, sows in the vaccine group received two doses of the vaccines through intramuscular injection in the neck area: the first dose was given 6 weeks prior to farrowing, and the second dose, 3 weeks before farrowing. Sows in the control group remained vaccinated against EC and CPC as described above (see Section 2.1). To ensure the blind administration of the product, the treatment dispenser was the only person aware of the correspondence between the study products and the assigned sow groups. The treatment dispenser was not involved in any other aspect of the study.

### 2.3. Clostridioides Difficile and Clostridium Perfringens Assessments

The presence of CD and CPA, along with other pathogens responsible for neonatal diarrhea in swine (e.g., EC, *Rotavirus*), was confirmed on both farms prior to the commencement of the corresponding studies (see Supplementary Materials Tables S1–S4). In Study A, several months before the start of the study (July 2020, October 2020, and April 2021), diarrhea samples from 30 non-treated piglets were collected using Fast Technology Analysis (FTA) Cards and sent to HIPRA DIAGNOS (Amer, Girona, Spain) for polymerase chain reaction (PCR) analysis using Enterocheck^®^, as previously reported [19]. In addition, seven non-treated piglets presenting diarrhea were euthanized, and their whole intestinal mass was sent to EXOPOL S.L. (Zaragoza, Spain) to be tested with a diagnostic panel including molecular (i.e., PCR) and microbiological assays.

In study B, a few months before the start of the study (October–November 2021), fecal samples were obtained from 11 diarrheic, non-treated, 1-to-7-day-old piglets and analyzed for toxins A and B from CD using a commercial enzyme immunoassay (RIDASCREEN^®^, R-Biopharm, Darmstadt, Germany), following the manufacturer’s instructions. CP was isolated and subjected to genotyping as previously described [20]. EC was isolated in agar MacConkey, and the presence of its virulence factors was assessed as in the study of Macêdo et al. [21]. Finally, the presence of rotavirus A, B, and C was evaluated by RT-PCR as previously described [22,23,24].

### 2.4. Study Variables

The variables collected in the study during the lactation period included: (1) the number of litters affected by diarrhea (i.e., having ≥1 piglet with diarrhea); (2) the number of piglets with diarrhea in each group; (3) the number of piglets treated with antibiotics against diarrhea in each group; (4) the pre-weaning mortality rate. Antimicrobial administration was allowed only after the third consecutive day of diarrhea in the litter. Thereafter, if necessary, all affected piglets could start antibiotic treatment simultaneously. Additionally, four growth performance-related variables were collected: (5) body weight (BW) at birth measured during the first 48 h, (6) ADWG between farrowing day and weaning day, (7) BW at weaning, and (8) number of underweight piglets at weaning. Due to differences in weaning times between the two studies, underweight animals were defined as those with a deviation of the body weight at weaning of 25% below the average weight of the population. In study A, this threshold was set at 4.65 kg, while in study B it was 4.1 kg.

### 2.5. Statistical Analyses

Categorical variables were presented as frequencies and percentages, and quantitative variables as mean and standard deviation (SD). Differences between groups were assessed through regression models: a linear regression model was used for continuous variables, and a logistic regression model was used for categorical variables. Data from the two farms (Studies A and B) were combined in a meta-analysis to enhance the study’s statistical power. Country was used as a fixed effect in the models used for the meta-analysis. BW at birth was used as a covariate in the models related to performance in both the meta-analysis and Study A, because BW at birth varied significantly between groups, specifically in these analyses (see Supplementary Materials Table S5). Results are presented as descriptives statistics from raw data and least squares means (LSM) differences from both models and 95% confidence intervals (95% CI). The significance threshold was set at a bilateral alpha value of 0.05. All statistical analyses were performed using R software (version 4.4.0; https://www.R-project.org, accessed on 20 December 2024).

## 3. Results

### 3.1. Description of Productive Parameters, Clinical Parameters, and Use of Antibiotics

Table 1 presents the descriptive statistics for the performance parameters of the two groups in each of the farms studied, as well as for the meta-analysis. Briefly, the average BW at birth per study ranged between 1.35 kg and 1.49 kg in the control group, whereas in the vaccination group, it ranged between 1.40 kg and 1.65 kg. The ADWG varied from 156.81 g/day to 175.29 g/day in the control group, while in the vaccination group, it ranged from 185.96 g/day to 191.59 g/day. BW at weaning ranged between 4.98 kg and 5.26 kg in the control group, compared to 5.41 kg and 6.11 kg in the vaccination group. The percentage of underweight piglets ranged from 25.2% to 36.5% in the control group, whereas in the vaccination group, it varied between 16.7% and 18.5%.

Table 2 presents the descriptive statistics for the clinical parameters and antibiotic use of the two study groups in each of the farms studied, as well as for the meta-analysis. The percentage of litters with at least one piglet with diarrhea before weaning per study ranged from 33.3% to 90.5% in the vaccination group and from 62.5% to 96.0% in the control group. The percentage of piglets with diarrhea before weaning varied between 18.9% and 25.3% in the vaccination group, compared to 27.9% to 37.1% in the control group. The percentage of piglets treated with antibiotics before weaning ranged from 13.3% to 21.2% in the vaccination group and from 13.7% to 43.3% in the control group. Lastly, pre-weaning mortality ranged between 5.48% and 5.65% in the vaccination group, while in the control group, it ranged from 4.95% to 7.82%.

### 3.2. Association Between Vaccination with Productive Parameters, Clinical Parameters, and Use of Antibiotics

The meta-analysis from the two studies with a total of 903 piglets farrowed by 66 sows showed that vaccination had a significant positive effect on all variables related to weight performance at weaning. BW at birth showed a statistically significant difference between groups in both the meta-analysis and Study A, and was therefore included as a covariate in the statistical models related to performance. In contrast, no significant differences were observed in BW at birth between groups for Study B (Supplementary Materials Table S5). The LSM analysis indicated a statistically significant increase in ADWG and body weight at weaning in the vaccine group for the meta-analysis and individual studies (Table 3). Specifically, the meta-analysis showed a LSM difference in ADWG between the control and vaccine groups of −14.5 g/day (95% CI: −22.60 to −6.50, *p* < 0.001), indicating higher weight gain in the vaccine group. Similarly, the LSM difference in BW at weaning showed lower performance in the control group, with an LSM difference of −0.33 kg (95% CI: −0.50 to −0.16, *p* < 0.001) compared to the vaccine group. Additionally, the percentage of underweight piglets at weaning was significantly lower in the vaccine group, with an LSM difference of 6.94% (95% CI: 1.64 to 12.20, *p* = 0.011) compared to the control group. The detailed results of the regression analyses are shown in Supplementary Materials Table S5.

Disaggregated results by study (A and B) showed similar results. In Study A, the ADWG was significantly higher in vaccinated piglets, with a LSM difference of −21.40 g/day (*p* < 0.001), leading to a 0.51 kg (*p* < 0.001) higher body weight at weaning. Additionally, the percentage of underweight piglets at weaning was 12.6% lower (*p* = 0.024) in the vaccine group (Table 3).

In Study B, similar trends were observed but with smaller effect sizes. The vaccinated piglets had significantly higher ADWG (LSM difference of −16.30 g/day, *p* = 0.002) and BW at weaning (LSM difference of −0.42 kg, *p* < 0.001). The percentage of underweight piglets at weaning was 6.77% lower (*p* = 0.036) in the vaccine group, demonstrating a beneficial effect of vaccination on weight performance (Table 3).

Table 4 presents the statistical associations for clinical parameters and antibiotic use. Overall, the results indicate that vaccination was associated with reduced prevalence of diarrhea and decreased antibiotic use in piglets across both individual studies and the meta-analysis. Specifically, the percentage of piglets with diarrhea before weaning was significantly lower in the vaccine group on the meta-analysis, with an LSM difference of 9.76% (95% CI: 4.06–15.50, *p* < 0.001). Likewise, the percentage of piglets requiring antibiotic treatment was significantly reduced in the vaccine group with an LSM difference of 6.09% (95% CI: 1.16–11.00, *p* = 0.016). However, differences in the percentage of litters with at least one piglet experiencing diarrhea and pre-weaning mortality were not statistically significant. The detailed results of the regression analyses can be found in Supplementary Materials Table S6.

Disaggregated results by study (A and B) showed similar results (Table 4). In Study A, the vaccine group had a lower incidence of diarrhea and antibiotic use compared to the control group, though not all differences were statistically significant. The percentage of litters with at least one piglet with diarrhea before weaning showed an LSM difference of 29.20% in the vaccine group compared to the control, but this difference was not significant (*p* = 0.206). The percentage of piglets with diarrhea showed an LSM difference of 11.8% in the vaccine group, suggesting a statistical trend, although this difference was not statistically significant (*p* = 0.051). However, antibiotic use was significantly reduced in the vaccine group, with an LSM difference of 22.1% (*p* < 0.001). Pre-weaning mortality showed no statistical differences between groups (*p* = 0.855).

In Study B, the vaccine group also showed improvements, particularly in reducing diarrhea. The percentage of piglets with diarrhea before weaning was significantly lower with an LSM difference of 9.00% (*p* = 0.007) in the vaccine group. However, the difference in litters with at least one piglet with diarrhea (LSM means difference of 5.52%) was not statistically significant (*p* = 0.463). Antibiotic use was similar between groups, with an LSM difference of 0.40% (*p* = 0.882). Pre-weaning mortality was slightly lower in the vaccine group (LSM difference of 2.17%), but this was not statistically significant (*p* = 0.273).

## 4. Discussion

The current study aimed to expand knowledge about the effect on pig health of vaccination against CD and CPA under field conditions. A previous study showed that the new vaccine against CD and CPA infection effectively reduced the incidence of diarrhea and antibiotic usage in piglets during the first 7 days of age [18]. In the current study, the vaccinated group had a lower percentage of piglets with diarrhea before weaning and a reduced percentage of piglets treated with antibiotics for diarrhea compared to the control group, further confirming its impact on diarrhea and antibiotic use in suckling piglets. Additionally, our study provides support for the positive effects on minimizing the negative impact of the disease on weight performance for the first time. In this sense, the new vaccine was significantly associated with increased ADWG during the suckling period and higher body weight at weaning in piglets farrowed by vaccinated sows compared to those farrowed by controls. The improved weight performance in the vaccine group also resulted in a decreased percentage of underweight piglets at weaning.

Furthermore, we observed that the percentage of sows delivering at least one suckling piglet with diarrhea did not show a statistically significant decrease following vaccination. A possible explanation for the lack of statistical significance may lie in the lower number of sows compared to the study by Gibert et al. [18], which may have reduced the statistical power of our analyses. Nevertheless, the overall percentage of piglets with diarrhea before weaning did show a statistically significant decrease compared with the control group in most of the populations analyzed, which is consistent with the findings of Gibert et al. [18]. Despite this decrease in the occurrence of diarrhea, it did not fully correlate with antibiotic usage, especially in study B. This observation suggests that factors other than antibiotic treatment may play a significant role in the incidence of diarrhea [4]. Overall, the clinical results indicate that vaccination of sows led to a reduced incidence of neonatal diarrhea in both farms, as expected.

Besides the incidence of neonatal diarrhea, the present study also evaluated the growth performance of piglets during the lactation period, showing improved growth parameters in the vaccine group compared with the control group. Positive associations between vaccination and these parameters were observed overall and in Studies A and B. This is the first study showing the beneficial effect on piglet growth of a vaccine against Neonatal diarrheas caused by CD and CP. As previously mentioned, the disease can be caused by different agents. It is interesting to note that the use of vaccination against the neonatal diarrheas caused by EC and CP also contributes to improving the productive performances of piglets [17], indicating that combined vaccination plans can provide a broad spectrum of protection, resulting in clinical and productive benefits.

Another significant finding was the reduced percentage of piglets treated with antibiotics in animals born from vaccinated sows in Study A and in the meta-analysis from Studies A and B. The use of antibiotics in food animals is a major factor involved in the dissemination of antibiotic-resistant bacteria [25], and interventions that limit antibiotic use are associated with a decrease in antibiotic-resistant bacteria in these animals [26]. Therefore, besides the positive economic impact of using fewer antibiotics, vaccination with the tested vaccine may also help to reduce antibiotic resistance and, consequently, might benefit human health. Another benefit of reducing antibiotic use in suckling piglets may reside in gut microbiota composition, which plays an important role in piglet health and nutrition and stimulates the development of the immune system [27]. In this regard, some studies have shown that antibiotic exposure in suckling piglets altered the intestinal tract in terms of gene expression and microbiota composition, leading to an unhealthy gut environment [28,29,30]. Besides, morbidity rates in piglets with clostridial infection can reach up to 100%, although the associated mortality is generally low, as most piglets recover despite having lower body weights [4,5,17]. In the present study, no significant differences in pre-weaning mortality rates were detected between groups, which aligns with previous findings.

Despite showing positive results for the vaccine, this work has additional limitations beyond those previously mentioned. First, the multifactorial etiology of swine neonatal diarrhea under field conditions may have interfered with the effects vaccine under evaluation (i.e., other causative agents of diarrhea may have dampened the observed effects of the vaccine). Additionally, a limitation of the meta-analysis lies in the differences between Studies A and B, especially at the time of weaning, which could affect the variables measured at weaning (e.g., number of underweight piglets at weaning, body weight at weaning, etc.). Moreover, some of the initially recruited sows had missing data on diarrheas, and three sows were excluded in the vaccination group, as well as one the control group due to dying, aborting, or not giving birth to any living piglets in each of the studies. Overall, the reduced sample size might have impacted the results, indicating that larger studies will be useful to confirm the vaccine’s effectiveness. Nevertheless, the results from Studies A and B for variables measured at weaning were similar and supported an increased weight at weaning for piglets from vaccinated sows.

To our knowledge, this study is the first to assess the efficacy of a commercial vaccine against CD and CPA in relation to weight performance. Our findings suggest that the vaccine can positively impact the percentage of piglets with diarrhea and the ADWG, leading to greater productivity and improved animal welfare. These are relevant observations considering that lactation and nursery are critical stages regarding the retardation of growth in swine [31] and that neonatal diarrhea has been related to a longer rearing period [14]. Finally, piglets from the vaccinated group were less likely to require antibiotic treatment, which is also relevant from a One Health perspective [32]. In addition, the characteristics of the studied farms regarding the incidence of neonatal diarrhea and CD and CPA detection were very similar to those of most European swine farms [33]. This makes our results likely applicable to other farms with these characteristics (i.e., recurrent neonatal diarrhea and positive diagnosis of CD).

## 5. Conclusions

The present study suggests that the new commercial vaccine against CD and CPA reduces the incidence of neonatal diarrhea and positively impacts the growth performance of suckling piglets.

## 6. Patents

X. Gibert is one of the inventors of the PCT patent application PCT/EP2018/065025, filed by HIPRA SCIENTIFIC S.L.U. on 7 June 2018 and published on 13 December 2018, disclosing vaccines comprising clostridium toxoids, including the Suiseng Diff/A product used in the present work.

## Figures and Tables

**Table 1:** Descriptive statistics of performance parameters.

	Group	N	Body Weight at Birth (kg), Mean (SD)	ADWG ^b^ (g/day), Mean (SD)	Body Weight at Weaning ^b^ (kg), Mean (SD)	Underweight Piglets at Weaning ^b^, *n* (%)
Meta-analysis (A + B) ^a^	Vaccine	446	1.48 (0.35)	189.79 (61.07)	5.63 (1.52)	80 (17.9)
	Control	457	1.38 (0.36)	171.03 (62.22)	5.05 (1.42)	126 (27.6)
Study A ^a^	Vaccine	138	1.65 (0.34)	185.96 (54.10)	6.11 (1.51)	23 (16.7)
	Control	96	1.49 (0.36)	156.81 (44.01)	5.26 (1.12)	35 (36.5)
Study B	Vaccine	308	1.40 (0.32)	191.59 (64.10)	5.41 (1.47)	57 (18.5)
	Control	361	1.35 (0.35)	175.29 (66.19)	4.98 (1.50)	91 (25.2)

^a^ These analyses excluded the data of eight piglets from the vaccine group and one from the control group from study A because they died before weaning. ^b^ Weaning was performed at 24 days after farrowing in study A and at 21 days after farrowing in study B. ADWG, average daily weight gain.

**Table 2:** Descriptive statistics of clinical parameters and antibiotic use.

	Group	Litters	Litters with ≥1 Piglet with Diarrhea BW ^b^, *n* (%)	Piglets	Piglets with Diarrhea BW ^b^, *n* (%)	Piglets Treated with Antibiotics BW ^b^, *n* (%)	Pre-Weaning Mortality ^b^, *n* (%)
Meta-analysis (A + B) ^a^	Vaccine	33 ^a^	23 (69.7)	447 ^a^	94 (21.0)	71 (15.9)	25 (5.59)
	Control	33 ^a^	29 (87.9)	455 ^a^	136 (29.9)	91 (20.0)	33 (7.19) ^c^
Study A	Vaccine	12	4 (33.3)	146	37 (25.3)	31 (21.2)	8 (5.48)
	Control	8	5 (62.5)	97	36 (37.1)	42 (43.3)	5 (4.95) ^c^
Study B ^a^	Vaccine	21 ^a^	19 (90.5)	301 ^a^	57 (18.9)	40 (13.3)	17 (5.65)
	Control	25 ^a^	24 (96.0)	358 ^a^	100 (27.9)	49 (13.7)	28 (7.82)

^a^ These analyses excluded the data of three litters from the vaccine group and one from the control group because their size was <nine piglets. ^b^ Weaning was performed at 24 days (study A) and 21 days (study B) after farrowing. ^c^ Total number of piglets included four extra piglets that died just after farrowing. BW, before weaning.

**Table 3:** Least squares mean (LSM) differences and 95% Confidence Intervals (95% CI) of performance parameters between control (C) and vaccine (V) groups.

		Body Weight at Birth (kg)	ADWG ^b^ (g/day)	Body Weight at Weaning ^b^ (kg)	Underweight Piglets at Weaning ^b^ (%)
Meta-analysis (A + B) ^a^	LSM Difference between C and V	−0.07	−14.5	−0.33	6.94
	95% CI	(−0.12–−0.03)	(−22.60–−6.50)	(−0.50–−0.16)	(1.64–12.20)
	*p*-value	0.001	<0.001	<0.001	0.011
Study A ^a^	LSM Difference between C and V	−0.15	−21.40	−0.51	12.60
	95% CI	(−0.25–−0.06)	(−34.00–−8.75)	(−0.82–−0.21)	(1.35–23.80)
	*p*-value	0.001	<0.001	<0.001	0.024
Study B	LSM Difference between C and V	−0.05	−16.30	−0.42	6.77
	95% CI	(−0.10–0.01)	(−26.70–−5.95)	(−0.66–−0.19)	(0.53–13.00)
	*p*-value	0.079	0.002	<0.001	0.036

^a^ These analyses excluded the data of eight piglets from the vaccine group and one from the control group from study A because they died before weaning. ^b^ Weaning was performed at 24 days after farrowing in study A and at 21 days after farrowing in study B. ADWG, average daily weight gain.

**Table 4:** Least squares mean (LSM) differences and standard error (SE) of clinical parameters and antibiotic use between control (C) and vaccine (V) groups.

		Litters with ≥1 Piglet with Diarrhea BW ^b^ (%)	Piglets with Diarrhea BW ^b^ (%)	Piglets Treated with Antibiotics BW ^b^ (%)	Pre-Weaning Mortality ^b^ (%)
Meta-analysis (A + B) ^a^	LSM Difference between C and V	12.90	9.76	6.09	1.44 ^c^
	95% CI	(−4.81–30.50)	(4.06–15.50)	(1.16–11.00)	(−1.75–4.63)
	*p*-value	0.149	<0.001	0.016	0.377
Study A	LSM Difference between C and V	29.20	11.80	22.10	−0.53
	95% CI	(−13.70–72.00)	(−15.40–23.70)	(10.20–34.00)	(−6.14–5.09)
	*p*-value	0.206	0.051	<0.001	0.855
Study B ^a^	LSM Difference between C and V	5.52	9.00	0.40	2.17 ^c^
	95% CI	(−9.19–20.20)	(2.58–15.4)	(−4.83–5.63)	(−1.64–5.99)
	*p*-value	0.463	0.007	0.882	0.273

^a^ These analyses excluded the data of three litters from the vaccine group and one from the control group because their size was < nine piglets. ^b^ Weaning was performed at 24 days (study A) and 21 days (study B) after farrowing. ^c^ Total number of piglets included four extra piglets that died just after farrowing, in the control group. BW, before weaning.

## Data Availability

The data presented in this study are available on request from the corresponding author due to intellectual property protection and confidentiality.

## Supplementary Materials

The following supporting information can be downloaded at: https://www.mdpi.com/article/10.3390/ani15091200/s1, Table S1: Molecular diagnosis of microorganisms in diarrhea samples of non-treated piglets in study A, N (%).; Table S2: Molecular and bacteriological diagnosis of neonatal diarrhea in the whole intestinal mass of non-treated piglets with diarrhea in study A; Table S3: Histological changes in the whole intestinal mass of non-treated piglets with diarrhea in study A (October 2020); Table S4: Molecular and bacteriological diagnosis of neonatal diarrhea in diarrhea samples of non-treated piglets in study B (October–November 2021), N (%). N = 11; Table S5: Regression analyses showing the associations between the vaccination and the variables related to growth performance; Table S6: Regression analyses showing the associations between the vaccination and the variables related to clinical parameters and antibiotic use.

## Abbreviations

The following abbreviations are used in this manuscript:

ADWG	Average daily weight gain
CD	*Clostridioides difficile*
CP	*Clostridium perfringens*
CPA	*Clostridium perfringens* type A
CPC	*Clostridium perfringens* type C
EC	*Escherichia coli*
FTA	Fast Technology Analysis
PCR	Polymerase chain reaction
RT-PCR	Reverse transcription polymerase chain reaction
SD	Standard deviation
