# Supplementary material for: Efficacy of a New Commercial Vaccine Against Clostridioides difficile and Clostridium perfringens Type A for Recurrent Swine Neonatal Diarrhea Under Field Conditions
Source: Animals (Basel). 2025 Apr 23;15(9):1200. doi: 10.3390/ani15091200 (PMC12070884; doi:10.3390/ani15091200)
Supplement: Supplementary file 1 [file animals-15-01200-s001.zip › animals-3500626-supplementary.pdf]

Supplementary Table S1. Molecular diagnosis of microorganisms in diarrhea samples of non-treated piglets in study A, *N (%).*

	**July 2020**	**October 2020**	**April 2021**	
	(N=6)	(N=10)	(N=12)	(N=2)
Clostridioides difficile, toxin A	4 (66.7)	8 (80.0)	12 (100.0)	1 (50.0)
Clostridioides difficile, toxin B	5 (83.3)	4 (40.0)	12 (100.0)	1 (50.0)
Clostridium perfringens, toxin alpha	6 (100.0)	10 (100.0)	12 (100.0)	2 (100.0)
Clostridium perfringens, toxin beta	1 (16.7)	0 (0.0)	0 (0.0)	0 (0.0)
Clostridium perfringens, toxin epsilon	0 (0.0)	0 (0.0)	0 (0.0)	0 (0.0)
Escherichia coli, F4	1 (16.7)	2 (20.0)	12 (100.0)	0 (0.0)
Escherichia coli, F5	1 (16.7)	1 (10.0)	0 (0.0)	0 (0.0)
Escherichia coli, LT	2 (33.3)	10 (100.0)	0 (0.0)	0 (0.0)
Escherichia coli, F6	1 (16.7)	4 (40.0)	12 (100.0)	0 (0.0)

Supplementary Table S2. Molecular and bacteriological diagnosis of neonatal diarrhea in the whole intestinal mass of non-treated piglets with diarrhea in study A.

	**July 2020**	**October 2020**		**April 2021**	
	(pooled samples of piglets 1, 2, and 3)	Piglet 4	Piglet 5	Piglet 6	Piglet 7
**Molecular diagnosis**					
Rotavirus type A	Negative	**Positive**	**Positive**	Negative	Negative
PEDV	Negative	Negative	Negative	Negative	Negative
TGEV	Negative	Negative	Negative	Negative	Negative
Eimeria spp.	Negative	Negative	Negative	NA	Negative
Cystoisospora suis	Negative	**Positive**	Negative	Negative	**Positive**
Clostridium perfringens	**Positive** (alpha and beta2 toxins)	**Positive** (alpha toxin)	**Positive** (alpha toxin)	**Positive** (alpha and beta2 toxins)	**Positive** (alpha and beta2 toxins)
Clostridioides difficile	**Positive**	Negative	**Positive**	**Positive**	**Positive**
Rotavirus type C	Negative	Negative	Negative	**Positive**	Negative
**Bacteriological diagnosis**	NA	*Streptococcus* sp.	Enterobacteriaceae; *Clostridium symbiosum*	*Clostridium perfringens;* Beta-hemolytic *Escherichia coli; Escherichia coli*	*Clostridium perfringens; Escherichia coli*

NA, not available; PEDV, porcine epidemic diarrhea virus; TGEV, transmissible gastroenteritis virus.

Supplementary Table S3. Histological changes in the whole intestinal mass of non-treated piglets with diarrhea in study A (October 2020).

	Piglet 4	Piglet 5
Small intestine	No macroscopic or microscopic lesions. Normal villus length. Semi-solid greenish content. Abundant medium-sized, Gram-positive bacilli in the content, without contact with the mucosa.	No macroscopic lesions. Absence of content. Histologically, a slight cutting of villi is observed, without other types of alterations in the epithelium or in the mucosa. Scarce presence of Gram-positive bacilli, without contact with the mucosa.
Colon	Significant mesocolon edema with no other macroscopic or microscopic lesions. Absence of content. Moderate presence of medium-sized, Gram-positive bacilli and small-sized, Gram-negative bacilli, without contact with the mucosa.	Moderate-to-marked mesocolon edema with no other macroscopic or microscopic lesions. Semi-solid, whitish content with Gram-positive, medium-sized bacilli and some small-sized, Gram-negative bacilli, occasionally in contact with the surface of the epithelium.
Lymph node	No macroscopic or microscopic lesions.	No macroscopic or microscopic lesions.

Supplementary Table S4. Molecular and bacteriological diagnosis of neonatal diarrhea in diarrhea samples of non-treated piglets in study B (October-November 2021), *N (%)*. N=11.

Clostridioides difficile A/B toxins	6 (54.5)
Clostridium perfringens	7 (63.6)
Escherichia coli	11 (100.0)[a]
Rotavirus (A, B, and C)	0 (0.0)[b]

[a]Isolated, but negative by polymerase chain reaction.

[b]The sample size was reduced to n=5 due to insufficient material.

Supplementary Table S5. Regression analyses showing the associations between the vaccination and the variables related to growth performance.

	Meta-analysis (A+B)				Study A				Study B			
	**Estimate**	**Std. error**	**T-value**	**P-value**	**Estimate**	**Std. error**	**T-value**	**P-value**	**Estimate**	**Std. error**	**T-value**	**P-value**
**Body weight at birth**												
Intercept	1.341	0.017	79.152	<0.001	1.492	0.036	42.004	<0.001	1.354	0.018	75.691	<0.001
Vaccination	0.074	0.023	3.209	0.001	0.154	0.046	3.322	0.001	0.046	0.026	1.758	0.079
Country	0.198	0.026	7.550	<0.001	-	-	-	-	-	-	-	-
**ADWG**[a]												
Intercept	99.751	8.694	11.47	<0.001	81.425	14.112	5.77	<0.001	175.290	3.645	48.095	<0.001
Body weight at birth	55.394	6.101	9.08	<0.001	50.540	8.894	5.683	<0.001	-	-	-	-
Vaccination	14.538	4.096	3.55	<0.001	21.385	6.412	3.335	<0.001	16.300	5.272	3.092	0.002
Country	-21.968	4.684	-4.69	<0.001	-	-	-	-	-	-	-	-
**Body weight at weaning**[a]												
Intercept	1.988	0.186	10.670	<0.001	1.954	0.339	5.770	<0.001	4.984	0.083	60.140	<0.001
Body weight at birth	2.205	0.131	16.868	<0.001	2.213	0.213	10.368	<0.001	-	-	-	-
Vaccination	0.330	0.088	3.758	<0.001	0.513	0.154	3.335	<0.001	0.422	0.120	3.516	<0.001
Country	0.087	0.100	0.8690	0.385	-	-	-	-	-	-	-	-
	**Estimate**	**Std. error**	**Z-value**	**P-value**	**Estimate**	**Std. error**	**Z-value**	**P-value**	**Estimate**	**Std. error**	**Z-value**	**P-value**
**Percentage of underweight piglets at weaning**[a]												

Intercept	2.063	0.364	5.667	<0.001	3.868	0.878	4.406	<0.001	-0.184	0.121	-8.937	<0.001
Body weight at birth	-2.401	0.280	-8.587	<0.001	-3.053	0.596	-5.127	<0.001	-	-	-	-
Vaccination	-0.439	0.172	-2.555	0.011	-0.766	0.339	-2.263	0.024	-0.399	0.190	-2.094	0.036
Country	0.698	0.199	3.504	<0.001	-	-	-	-	-	-	-	-

[a]Weaning was performed at 24 days after farrowing in study A and at 21 days after farrowing in study B. ADWG, average daily weight gain.

Supplementary Table S6. Regression analyses showing the associations between the vaccination and the variables related to clinical parameters and antibiotic use.

	Meta-analysis (A+B)				Study A				Study B			
	Estimate	Std. error	Z-value	P-value	Estimate	Std. error	Z-value	P-value	Estimate	Std. error	Z-value	P-value
**Percentage of litters with ≥1 piglet with diarrhea BW[a]**												
Intercept	3.298	0.800	4.122	<0.001	0.511	0.730	0.699	0.484	3.178	1.021	3.114	0.002
Vaccination	-1.106	0.765	-1.445	0.149	-1.204	0.953	-1.263	0.206	-0.927	1.263	-0.734	0.463
Country	-2.845	0.766	-3.716	<0.001	-	-	-	-	-	-	-	-
**Percentage of piglets with diarrhea BW[a]**												
Intercept	-0.942	0.110	-8.534	<0.001	-0.527	0.210	-2.509	0.0121	-0.948	0.118	-8.046	<0.001
Vaccination	-0.521	0.157	-3.315	<0.001	-0.553	0.284	-1.951	0.051	-0.506	0.189	-2.687	0.007
Country	0.397	0.170	2.332	0.020	-	-	-	-	-	-	-	-
**Percentage of piglets treated with antibiotics BW[a]**												
Intercept	-1.675	0.133	-12.567	<0.001	-0.270	0.205	-1.316	0.188	-1.842	0.154	-11.976	<0.001
Vaccination	-0.435	0.181	-2.403	0.016	-1.041	0.288	-3.616	<0.001	-0.034	0.229	-0.149	0.882

Country	1.083	0.184	5.878	<0.001	-	-	-	-	-	-	-	-
**Pre-Weaning Mortality[a] (%)**												
Intercept	-2.753	0.321	-8.568	<0.001	-2.955	0.459	-6.442	<0.001	-2.467	0.197	-12.533	<0.001
Vaccination	-0.244	0.276	-0.884	0.377	0.107	0.585	0.183	0.855	-0.349	0.318	-1.097	0.273
Country	0.245	0.326	0.749	0.454	-	-	-	-	-	-	-	-

[a]Weaning was performed at 24 days after farrowing in study A and at 21 days after farrowing in study B. BW, before weaning.
